# The Longitudinal Association Between Organized Leisure Sports and School-Related Social Well-Being

**DOI:** 10.3390/healthcare14193288

**Published:** 2026-10-02

**Authors:** Sanne Lund Clement, Rasmus Lind Ravn, Rasmus Juul Møberg, Lotte Stausgaard Skrubbeltrang

**Affiliations:** 1 Department of Society and Politics, Aalborg University, Kroghstraede 7, 9220 Aalborg East, Denmark; clement@society.aau.dk (S.L.C.); rjm@society.aau.dk (R.J.M.); 2 Department of Social Education, University College of Northern Denmark, Mylius Erichsens Vej 137, 9210 Aalborg South East, Denmark; lst@ucn.dk

**Keywords:** youth, well-being, sports participation, leisure, augmented inverse probability weighting, longitudinal design, observational study

## Abstract

**Background/Objectives**: Previous research has found positive associations between physical activity and leisure-time sport participation and subjective well-being among children and adolescents. However, most of this evidence is cross-sectional. In this study, we add to this discussion by examining the hypothesized positive longitudinal association between participation in organized leisure sports and subsequent school-related well-being. **Methods**: We utilize different data sources: (1) pooled three-wave panel data for fifth- to ninth-grade pupils on sports participation, (2) register data on socio-economic background, and (3) the National Danish Well-Being Survey (initial sample size of 1210 pupils). We compile a longitudinal dataset with a distinguishable temporal ordering of exposure (participation in organized leisure sports), which is measured prior to the outcome (school-related well-being). In the statistical analyses, we employ augmented inverse probability weighting to minimize observed confounding. **Results:** Adjusting for the major confounders discussed in the literature, we find that participation in organized leisure sports is associated with higher subsequent school-related well-being levels of pupils (n = 998). The association was 0.18 (CI = 0.091–0.268) on a scale of 1–5, corresponding to a 4.4 percent increase, and a Cohen’s d of 0.31. **Conclusions**: Leisure-time sports participation was longitudinally associated with greater subjective well-being in a non-clinical population, with potential benefits extending to the school context. Further research using designs capable of strengthening causal inference is warranted.

## 1. Introduction

In recent years, increasing attention has been paid to children’s and adolescents’ social and mental well-being. Although most young people report high levels of well-being, evidence suggests an overall decline, particularly during adolescence [1,2,3,4,5,6,7]. This declining trend is highly concerning due to its adverse implications for education, employment, and long-term health [3,4,8,9].

The recent literature emphasizes the importance of social relations and leisure activities [10,11,12], particularly physical activity and organized sports. Physical activity (PA) is associated with improved health and aspects of subjective well-being [13,14,15,16], while participation in organized sports is linked to higher quality of life and well-being [17,18,19,20,21]. Organized sports also foster social participation, leadership, and social capital [22], and engagement in social communities correlates positively with social inclusion [23,24,25,26,27].

Most studies show strong correlations between leisure-time sport participation and young people’s well-being. While there are exceptions, most of the research is dominated by cross-sectional studies, as a recent review establishes [21]. While the benefits of physical activity for well-being are well established through reviews and meta-analyses of the literature [28], organized sports participation is inherently different from pure physical activity. While physical activity is a key aspect of sport, organized sports participation occurs in a *social* context, in which participants interact with peers and form social relationships and build social capital [21,22]. A recent meta-analysis explores the longitudinal relationship between youth sports participation and physical activity, health and wellbeing [29]. They find a low- to medium-sized positive relationship.

In our review of the literature, we have not been able to identify a single study examining the link between leisure-time sport and well-being within a school context. The study presented in this article seeks to contribute to the literature by examining if such an association exists.

Below, we outline why we theoretically expect an association to exist. In essence, we expect a spillover from leisure-time activities to well-being in a school context. This is theoretically interesting, as this could be a first step towards illustrating how leisure aspects of life outside of a school context have implications for the perception of time in school. Research consistently highlights social relationships as central to well-being both generally and within a school context [30,31,32,33].

A sense of belonging and acceptance at school is important for adolescents’ sense of self-worth and overall satisfaction with life [33]. Data from OECD’s surveys among children and adolescents in all OECD countries show a strong relationship between the likelihood of reporting low satisfaction with life and feeling like an outsider at school [34].

In Denmark, where our study is situated, more than 80 percent of children between the ages of 7–15 participate in sports or exercise [35]. In total, 82 percent of those who participate in sports do so in a sports association [35]. In addition, most leisure-time sport activities are organized in local organizations and sports associations. The majority of children who are active in sports are very likely to engage in sports with peers from their own school or class. Significant overlap between the social networks at sport and at school thus exists for many children. This is the main reason why we theoretically expect a rather direct, though still indirect relationship between sports participation and social well-being at school. Additional explanations for an association between participation in organized leisure-time sports might focus on the family environment or personality traits. Children experiencing a positive family environment might be more prone to participate in sport and benefit from participation, and it might also reinforce well-being in school. Likewise, extroverts might crave the social interactions associated with both sports and school.

The knowledge gap that this study seeks to bridge is the fact that the possible link between leisure-time sports participation and well-being in school has been overlooked in the existing literature.

We therefore pose the following research question and hypothesis:

*Is participation in organized leisure-time sports longitudinally associated with higher school-related social well-being for children and adolescents in the 5th* *–* *9th grades?*

## 2. Materials and Methods

### 2.1. Study Design and Procedures

Our study utilizes three sources of data to explore whether participation in organized leisure-time sports is associated with school-related social well-being (SRSW): longitudinal panel survey data, various longitudinal register data from Statistics Denmark, and data from the Danish National Well-Being Survey collected at all Danish lower secondary schools, which is provided by ‘the Agency for IT and Learning’ under the Danish Ministry of Children and Education.

The survey data consists of three waves of surveys collected among a panel of 5th–8th grade pupils from 11 different public schools in a small and rural Danish Municipality. All three surveys focused on examining leisure-time sports participation among the pupils and included additional questions about well-being, family and friends, motives for participation, etc. Written consent to participate in the study was obtained by the parents of all pupils included in the study prior to collecting the data. This included the use of the Danish personal identification number, allowing us to merge the survey data with the register data and the National Well-Being Survey (confer below).

### 2.2. Participants

The first wave was conducted at the beginning of April 2021. All pupils with their parents’ consent to participate (1210 pupils in fifth to eighth grade) received a unique link to the online questionnaire, with instructions that the survey would be completed during school time in their classroom at a specified time. The teachers were provided with a guided version of the questionnaire in advance. Unfortunately, not all teachers and schools allocated the time or prepared for the survey, and only 534 pupils completed the questionnaire. This meant that half of our study population did not get the opportunity to be part of the panel in the first wave. While this could be regarded as systematic non-response based on school and class, we interpret this as the panel not being fully established during the first round. The panel was expanded to include these schools and classes in the second wave. Before the second wave in October 2021, we scheduled meetings with each class at every school, where we presented the project to the pupils (now 6th to 9th grade), and they completed the questionnaire while we were present. In wave two, we had consent from the parents of 1334 pupils, and 930 of them completed the questionnaire. In wave three, conducted in May 2022, we arranged an online meeting with all schools at the same time, where we presented the third questionnaire, and they were able to ask questions before answering. The link was sent to 1224 pupils (6th to 9th grade), and 740 completed the questionnaire.

Table 1 below shows the distribution of responses in the three waves.

In the first wave of surveys, the pupils were in fifth to eighth grade and thus were aged 10–15 years old. The second and third waves were conducted at the beginning and the end of the following school year, respectively, when the pupils were in 6th to 9th grade. In all three waves, the panel had a fairly equal distribution of boys and girls, and we tracked these individuals over time.

For handling missing data, we used complete-case analysis. Missingness primarily arose due to organizational factors (teacher prioritization of participation) and student absence from school on the day of data collection. Although the missing data mechanism cannot be established with certainty, we have no evidence suggesting systematic associations between missingness and the key analytical variables. For detailed information on the participant flow and how we reach our analytical sample from the initial sample, please see Figure S1 in the Supplementary Materials. In addition, these key variables were included in the AIPW (socio-economic and demographic variables), which may alleviate possible selection biases.

However, if non-attendance is related to lower levels of school-related well-being, this would lead to an artificially higher average of well-being than the true average. Potentially, this could result in the association between sports participation and well-being being attenuated, potentially leading to more conservative estimates of the relationship.

### 2.3. Measures

The connected register data provided by Statistics Denmark consists of various registers containing information on parental employment and unemployment, parental educational attainment, family composition, and various other background characteristics for all individuals in the panel.

The official Danish National Well-Being Survey is conducted once a year, and all pupils from 0th to 9th grade complete the survey between January 20th and March 20th every year, but COVID-19 restrictions delayed data collection during our period of observation. Pupils from 0th to 3rd grade receive a much shorter and simpler version of the questionnaire, and pupils from 4th to 9th grade receive a different and longer version [36].

The dependent variable, school-related well-being, is measured using the Danish National Well-Being Survey as an instrument. It explores four dimensions of well-being, of which we focus on the social well-being dimension. The other three dimensions are “academic well-being”, “support and inspiration”, and “classroom noise and punctuality of teachers”. As we are examining a potential spillover from the leisure to school contexts, we focus only on the social well-being dimension. The “social well-being” indicator includes a total of 10 questions about the pupils’ perceptions of belonging in the school, the classroom, and as part of the group, and questions about bullying and the feeling of security. The 10 questions included are as follows:

(1) Are you happy at your school?
(2) Are you happy in your class?
(3) Do you feel lonely?
(4) Are you afraid of making a fool of yourself at school?
(5) How often do you feel safe at school?
(6) Have you been bullied this school year?
(7) I feel that I belong at school.
(8) Most of the pupils in my class are friendly and helpful.
(9) The other pupils accept me for who I am.
(10) I like recess at school.

Questions 1–7 have the response categories “very often–often–sometimes–rarely–never–I don’t want to answer”, and questions 8–10 have the response categories “completely agree–agree–neither agree nor disagree–disagree–completely disagree–I don’t want to answer”.

We do not include the other three dimensions of school well-being because we do not expect to find a link between organized leisure-time sports participation and these dimensions. Questions on these dimensions focus on whether the toilets and the classrooms are tidy, if the teacher arrives on time in class, if the teacher is helpful, whether the respondent does well in school and makes academic progress, noise in the classroom, and the quality of indoor and outdoor areas at school. Theoretically, we do not expect to find a link from any such questions.

The Ministry and the Agency provide an official guide for constructing approximately continuously scaled indices for research purposes. We have followed the official guide to construct the social well-being index, which we use as the dependent variable in our analyses. The index is constructed using questions measuring happiness, sadness, loneliness, bullying, et cetera, and it ranges from 1 to 5, including decimals. We use data from the 2022 National Well-Being Survey, which was also collected in the spring of 2022, coinciding with the last round of our panel survey data. We conducted a Cronbach’s alpha test to check the internal reliability of the index before constructing it. This test yielded a value of 0.87, suggesting a high degree of reliability suitable for scale construction.

As mentioned, the dependent variable does not examine the pupil’s well-being specifically related to their leisure-time activities, and the questions are framed in the school context. Nevertheless, the items and the well-being survey are the nationally agreed-upon measures that all Danish pupils are required to complete. The survey items were designed by a group of expert researchers to assess school well-being. The school-related social well-being index overlaps with and differs from other widely used measures of subjective well-being. For instance, it includes loneliness, similar to the psychological well-being dimension of KIDSCREEN-27, and happiness, as found in the WHO-5 and the Strengths and Difficulties Questionnaire (SDQ). In the well-being survey, however, happiness is explicitly tied to the pupil’s experiences at school or within the class, a perspective also reflected in KIDSCREEN-27. Bullying experiences are likewise included, as in the SDQ.

At the same time, the well-being survey contains several items that distinguish it from other well-being scales. Questions such as “Are you afraid of making a fool of yourself at school?” and “I like recess at school” are unique to this survey and are generally absent from alternative measures. Despite these differences, the SRSW dimension of the well-being survey shows substantial overlap with established measures of psychological well-being.

### 2.4. Data Analysis

To test if there is a longitudinal association between organized leisure-time sports and social well-being in school, we pool answers from the three rounds of surveys (T1, T2 and T3) and categorize respondents based on their latest status as either non-participants (sports inactive) or sports participants. We pool the answers to maximize the number of observations used in our analyses. The respondents whose latest status is ‘inactive’ constitute our control group, and sports participants constitute our treatment group.

Analyses were conducted using Stata 17.0. We used augmented inverse probability weighting (AIPW), a method that has recently gained popularity in the social sciences, particularly in studies of emerging adulthood [37]. AIPW builds on inverse probability weighting (IPW), with both methods belonging to the broader category of statistical matching or probability-weighting techniques. IPW involves three key steps. First, propensity scores are calculated, representing the probability of receiving treatment (in our case, a sports participant) based on the model’s covariates. Next, weights are assigned to ensure that individuals in both the treatment and control groups have an equal likelihood of receiving treatment. Finally, these weights are applied to correct for observed confounding, and the results are compared against the outcome variable to estimate the outcome.

AIPW introduces an additional step to this process. After the weights are applied, a regression analysis is conducted to further adjust for any remaining imbalances in the weights. In AIPW, the initial three steps are known as the treatment model, while the final step is referred to as the outcome model. We use a logit model for the treatment model, since treatment is binary (active in sports or not), and a linear regression model for the outcome, since the index is approximately continuous.

In essence, this means that only one of either the treatment model or the outcome model has to be correctly specified in order to yield correct estimates. This entails that the method has the property of double-robustness [38]. Reformulated, the outcome model can be misspecified, and the results will still be valid insofar as the treatment model is correctly specified. AIPW models adjust for observed confounding. To produce estimates for causal effects, there must be no unobserved confounding, which is not the case for our analyses. Our contribution to the literature is thus limited to longitudinal associations. Furthermore, AIPW analyses rest on the overlap and positivity assumption. Individuals must have a non-zero probability of receiving ‘treatment’. In our case, participating in sports or not. For our analyses, there is no indication that these assumptions are violated. Please see Figure S2 in the Supplementary Materials for an overlap plot.

In building our statistical model, we choose the major covariates that are known to be associated with participation in sports and organized leisure-time activities and well-being. Rephrased, we include variables that are known to be associated with ‘receiving treatment’ (participating in sports) and the outcome (well-being) to minimize confounding.

To guide our covariate selection, we briefly review the literature on factors influencing sports participation and well-being, respectively. We do this to be able to select the most appropriate covariates for our statistical analyses.

An abundance of research focuses on determinants of sports participation, in other words, factors associated with being active in sports. The demographic–economic model figures prominently in this literature and carries a great deal of explanatory power [39,40,41]. The demographic–economic model is derived from findings from empirical research, and, in essence, the model highlights that socio-demographic and economic factors influence the likelihood of participating in sports. A number of key determinants have been identified in the literature, and they consist of gender, age, ethnic background/migration, income, and human capital in the form of educational attainment. Briefly summarized, this strand of research finds that boys are more likely to participate than girls [42,43,44,45,46,47], and participation declines with age, especially during adolescence [41,43,47,48]. Individuals with migrant backgrounds participate less frequently, which is typically attributed to socio-economic and cultural barriers [49,50].

Higher income and parental employment increase participation, particularly for adolescents [43,44,51,52], while lower parental education is associated with reduced participation [7,19]. Family structure also matters, with lower participation among children from non-nuclear or larger households [18,53].

As with the literature on determinants of sports participation, there is also a vast amount of research examining factors related to subjective well-being. Many of these factors also broadly fall within the demographic–economic model.

Subjective well-being declines with age, especially among girls [4,7,54,55,56,57]. For both children and adults, having a migrant background is also a factor that, on average, is related to having lower levels of subjective well-being compared to not having a migrant background [58,59].

As was also the case with sports participation, income and socio-economic status are strong determinants of well-being [10]. For children and adolescents, parental employment and unemployment are related to increased and decreased well-being, respectively [60,61,62]. Furthermore, parental disability is also known to influence the well-being of children [63].

As already illustrated, parental educational attainment is also strongly related to offspring well-being, with children of parents with low educational attainment being more likely to suffer from low levels of subjective well-being [64,65].

Lastly, family composition and family structure are also highly related to child well-being outcomes. For instance, children from non-nuclear families and single-parent families exhibit low well-being levels [65].

In addition to the factors mentioned above, we also include a variable measuring prior sports participation and a variable measuring earlier levels of school-related social well-being to account for prior experiences.

Due to our rather small sample size, we include only the covariates the existing literature has highlighted as the most important factors for sports participation and well-being. We have, nevertheless, also carried out robustness checks with more covariates in the model. The results, however, remain qualitatively the same. For parsimonious reasons, we settled on the simpler statistical model, but we also show results with the inclusion of prior levels of social well-being to ensure transparency and to check if our results are robust.

The estimation equation for our analyses is:

$${\bar{ATE}}_{AIPW}=\frac{1}{n}\sum_{i=1}^{n}\left[\frac{Sport_{i}\left(Well-being_{i}-{\overset{ˆ}{m}}_{1}\left(X_{i}\right)\right)}{\overset{ˆ}{e}\left(X_{i}\right)}-\frac{\left(1-Sport_{i}\right)\left(Well-being_{i}-{\overset{ˆ}{m}}_{0}\left(X_{i}\right)\right)}{1-\overset{ˆ}{e}\left(X_{i}\right)}+{\overset{ˆ}{m}}_{1}\left(X_{i}\right)-{\overset{ˆ}{m}}_{0}\left(X_{i}\right)\right.$$

X is a vector of covariates measured at T − 1 (the year prior to the survey): well-being, own employment (measured in hours), mother’s employment (measured in hours), father’s employment (measured in hours), family composition, mother’s receipt of disability pension, father’s receipt of disability pension, gender, year of birth, mother’s educational attainment, father’s educational attainment, number of children in the household, country of origin/migrant background, prior sports participation. To ensure transparency, we reiterate the temporal ordering of our data. All included confounders are measured at the end of 2020 (for instance, information on parental educational attainment and family composition) or as accumulated throughout the year (employment is measured as the total number of working hours in 2020). Our exposure variable is measured three times (spring 2021, autumn 2021 and spring 2022), and the outcome variable is measured in spring 2022. As mentioned, we pool the exposure variable and use the last observed activity status for a respondent.

We considered other statistical methods besides AIPW for our analyses. These included fixed-effects regressions and mixed models. While these models are often used for longitudinal data, their advantages are less relevant in the present study. First, exposure was pooled across measurement occasions to ensure sufficient statistical power, thereby reducing the relevance of approaches that primarily exploit within-person temporal variation. The disadvantage of pooling the exposure variable is that within-person change, duration of participation, and temporal ordering of sports participation are not captured in our analyses. The panel expansion and attrition across waves, reducing the number of repeated observations, limit the use of panel data methods. Secondly, because exposure status showed limited within-person variation over time, a fixed-effects approach would rely on a relatively small number of individuals who change status between active and non-active. This results in potentially limited efficiency and possibly imprecise estimates. Of 301 sports active respondents in Wave 1, 258 remain active in Wave 2 (86%), and of 397 sports active respondents in Wave 2, 359 (90%) remained active in Wave 3. An additional reason for our choice is that our own survey on sports participation (Waves 1 to 3) unfortunately does not measure many of the relevant confounders across waves, entailing that we can only observe within-individual changes in the exposure, participation in leisure sports. This further limits the utility of such alternative panel data models. Furthermore, the register data and the National Well-being Survey consist of data from different points in time (at the end of each year), complicating such analyses even further, with difficulties in getting the temporal alignment correct. Weighing the advantages and disadvantages of using alternative methods on our specific data against each other, we decided to use AIPW.

## 3. Results

In this section, we start out by presenting descriptive statistics for the readers to familiarize themselves with the data and the relationship between sports participation and well-being. In Table 2 below, we start out by presenting the latest available activity status of our respondents and the mean value of the well-being index before and after our own survey focusing on sports participation was conducted.

First of all, Table 2 shows the share of respondents who are active in sport. This is the case for the vast majority, with almost three-quarters being sports participants. In relation to sports participation, we shall also provide some additional information not shown in the table. For instance, among the sports-active group, 63% participate in a team sport and 37% in an individual sport (not shown in a table). In addition, the most popular sports among our respondents are football/soccer (roughly 30%), handball (roughly a quarter), badminton (roughly 10%), gymnastics (roughly 10%), and horseback riding (also roughly 10%).

Returning to the results of Table 2, there is a tendency in relation to well-being among our respondents. Those who were not active in sport during our period of observation had lower levels of well-being on average before our surveys of sports participation (a difference in the mean of roughly 0.2 points on the five-point scale). The sports inactive group also had lower well-being levels after our surveys of sports participation (also a difference of roughly 0.2 points). This is indicative of a relevant issue. First, those with reduced well-being are less inclined to participate in sports and thereby less likely to reap the potential benefits of participation. In other words, there is strong self-selection into sports, and an issue of reverse causality may arise. We have explored this in Table S1 in the Supplementary Materials using a logistic regression analysis with sports participation as the dependent variable and several control variables back in time. The regression shows a statistically significant relationship between well-being in 2020 and sports participation in 2021/2022, highlighting the above-mentioned issue of self-selection. This suggests the need to take into account prior sports participation in the statistical model.

Below, we present our AIPW estimates in Table 3.

In Table 3 above, we outline our AIPW estimates. To ensure transparency, we also show the covariate balancing before and after weighting in Tables S2 and S3 in the Supplementary Materials. Tables S2 and S3 show standardized differences and variance ratios before and after weighting. In essence, the tables are used to inspect whether AIPW analyses effectively minimized the observed differences between our control group (those who are not active in sport) and the treatment group (the sports-active group). Small values (close to a value of zero) in the weighted standardized differences column indicate a high degree of balance after weighting. Similarly, values close to 1.0 in the weighted variance ratio imply a good balance. As can be seen from the tables in the Supplementary Materials, a good balance was achieved on observed confounders. We also conducted a formal overidentification test. The test attempts to reject the null hypothesis that covariates are balanced. The results were a χ^2^(14) = 5.94 and *p* = 0.97, clearly indicating that the null hypothesis cannot be rejected, suggesting that covariates were balanced.

The first row in Table 3 estimates the difference in subjective social well-being between sports participants and the group of non-participants. We find a positive and statistically significant adjusted association of a 0.18-point difference on a scale from 1 to 5. Sports participation is associated with 4.4 percent higher (calculation based on the total mean of 4.09) levels of school-related social well-being. Standardizing this coefficient yields a Cohen’s d of 0.31. Using the rules of thumb for interpreting Cohen’s d as a frame of reference, the strength of association is small to medium-sized. The association is roughly of the same magnitude as effect sizes found in meta-analyses on the subject. A meta-analysis on the outcomes of youth sport participation compared to non-participation reports a Cohen’s d of 0.23 on health and well-being [28], and a meta-analysis on outcomes of physical activity in healthy individuals reports a Cohen’s d of 0.34 on subjective well-being [29]. The practical implications of these results are discussed in the Section 4.

As evident from Table 1, our first wave suffered from a low response rate, and the panel was effectively expanded from Wave 2. The second row is a robustness analysis to check the impact of this attrition. The analysis included only respondents who did not participate in the first wave. The results are still statistically significant and of a similar level (slightly lower) as the results in the first row. The impact of this attrition is very modest.

The third row shows similar results as in the first row—the difference being that we have included prior well-being levels in the model. The estimated association is of a similar magnitude and still statistically significant. However, the number of observations used for the AIPW analysis drops from 998 to 586. This drop is caused by the fact that 412 of our respondents have not answered the National Well-Being Survey of 2019. The main reason for this drop is that those who were in the fifth grade in Wave 1, and the sixth grade in Waves 2 and 3, were in the third grade when the National Well-Being Survey of 2019 was conducted. This was the case for 294 of our respondents. The full well-being survey is only conducted for grades 4th through 9th. We are thus dealing with a subgroup that excludes the lowest grade of our study population. In this subgroup analysis, we still find a positive and significant association, which is also robust when taking into account past well-being levels.

The fourth row consists of an additional robustness test to check for exposure misclassification. It consists of a subgroup analysis including only respondents who answered the third wave of our survey. In addition, for this analysis, respondents who experienced a change in their sports participation status (for instance, from being sports active to inactive or the other way around) at any point during our period of observation were excluded from the analysis. This could be from either Wave 1 to Wave 2, from Wave 2 to Wave 3, or from Wave 1 to Wave 3. In essence, the subgroup consists of respondents who reported that they were either sports active or sports inactive in all the waves they answered. The results show a positive and highly significant association for this subgroup. This robustness analysis strengthens the notion that our results are not severely biased due to exposure misclassification.

### Exploratory Analyses

To make further use of our panel survey data on sports participation, we also carried out several exploratory analyses using only respondents who have answered all three surveys. We did this to examine whether prolonged exposure to organized sports is related to higher levels of well-being. These results are presented in Table 4 below.

The results in Table 4 above are compared to a group who were neither active in Waves 1, 2, or 3 (n = 45), that is, the whole of our observation period. Using these, the association is estimated for two groups. Those who were active in 1–2 waves (n = 46) and those who were active in all three rounds (n = 132). Because of the small number of observations used in this analysis, the results should be interpreted with much caution. Keeping this in mind, it can be seen from the table that there is a significant and positive association in all three waves (prolonged exposure/participation). For those with less exposure (1–2 waves of being active in sports), the association is not statistically significant, but the estimate is positive and one-third smaller than for those who were sports active throughout our period of observation. As mentioned, the exploratory analyses on prolonged exposure are underpowered and should be interpreted with caution. Performing analyses with multiple covariates on a small number of observations (223) might introduce overfitting. Overfitting in propensity score models often reveals itself through a failure to balance covariates or extreme weights. To check for this, we have produced an overlap plot available in Figure S3 in the Supplementary Materials. The propensity scores showed acceptable overlap. We also conducted a formal overidentification test to evaluate covariate balance for this subgroup analysis. Results were a χ^2^(13) = 6.04 and *p* = 0.94 comparing ‘no sport’ with sport in 1–2 waves, and a χ^2^(14) =2.67 and *p* = 0.99 comparing ‘no sport’ with sport in all waves. This clearly indicates that the null hypothesis cannot be rejected, suggesting that covariates were balanced. This suggests that the analysis in question does not suffer from overfitting.

The second half of Table 4 presents an additional exploratory analysis of prolonged exposure. It includes respondents who answered the third wave of the survey and at least one more wave. Like the last row of results in Table 3, it also exclusively includes respondents who did not experience a change in sports participation status throughout the period of observation. In essence, the exposure group consists of respondents who answered Wave 3 of our survey and were sports active in two or three waves, and the comparison group consists of respondents who were sports inactive in two or three waves. The results based on this analysis indicate a significant positive association (a coefficient of 0.24).

Overall, our results suggest that there is a positive relationship between organized leisure-time sports participation and school-related social well-being.

## 4. Discussion

Using a combination of panel survey data, register data, and National Well-Being Survey data, we have investigated whether organized leisure-time sports participation is associated with greater school-related social well-being among Danish children and adolescents in the fifth–ninth grade. By applying augmented inverse probability weighting to our longitudinal data, we believe we contribute to the literature on longitudinal associations between participation in organized leisure-time sports and well-being.

We find a statistically significant and positive relationship between sports participation and subsequent school-related social well-being. The Cohen’s d of 0.31 is similar to associations found in meta-analyses on related subjects [28,29]. The association persists even when taking prior levels of well-being and prior sports participation into account. Our analyses thereby contribute to the debate on the potential benefits of sports participation besides the health-related advantages of being physically active. Fluctuations and absolute levels of school-related social well-being have practical importance and real-world implications. The associations between school-related social well-being, specifically, and subsequent youth development outcomes and milestones have received limited scholarly attention. However, a systematic review, findsthat mental health and general subjective well-being are valid predictors of future NEET status among young people [9].

Our results furthermore showed that self-selection of leisure-time sports occurs. This also implies that although we observe the anticipated relationship, we cannot claim a causal relationship based on our findings.

However, existing studies indicate that sports participation can improve well-being among vulnerable youth or those experiencing serious challenges, provided that interventions are specifically tailored to address the needs of this target group. In an intervention study by Super et al. [20] focusing on vulnerable adolescents, a positive association was identified between participation in the intervention and measurable outcomes such as pro-social behavior, subjective health, well-being, and sense of coherence. In a systematic review of programs targeting socially vulnerable youth from 2017, Hermens and colleagues found that sports programs have the potential to make a positive difference for the development of what they refer to as life skills that can enhance well-being [66].

Despite the methodological advantages of our approach, there are a number of limitations to our study. First, we have not overcome causal inference issues, which means we cannot claim causality. Second, our study is prone to self-selection. The issue of self-selection might not only indicate reverse causality but can also blur the results of well-being as the dependent variable. Nevertheless, young people who drop out of sports might constitute a special group who do not benefit from sports participation in relation to social well-being. If this is the case, it would imply that we overestimate the association. Third, we are most likely dealing with an issue of unobserved heterogeneity. While our control and treatment groups are similar on observed characteristics after applying the statistical methods, they might still differ on factors relevant to both sports participation and well-being that we have not measured, nor could we measure. This could entail both an upward and a downward bias. Fourth, related to the concept of unobserved heterogeneity, there is still the potential risk of omitted variable bias, implying potential residual confounding. One such omitted variable could be personality traits of respondents, or screen time, which has been linked to decreased well-being. Some young people with certain personality traits might tend to *not* participate in sports and be better off for it in relation to their social well-being (for instance, introverts who might experience fatigue from being social with others). This could potentially cause a downward bias, suggesting that our estimates could be somewhat conservative. Fifth is the matter of external validity. Our study is situated in a Danish municipality. Sports participation through membership in local non-profit sports associations is high. Our results should only be generalized to similar contexts. Sixth, the panel expansion between Wave 1 and Wave 2 entailed substantial attrition.

Seventh, having adopted the use of augmented inverse probability weighting in our study offers the advantage of the double robustness property. The disadvantage of the approach is the difficulty of modeling temporal aspects of panel data into the analyses. A related limitation is the use of pooled data on the latest participation status, since it limits conclusions about within-person change, duration of participation, and temporality. This is a limitation of our study, which could be handled better by, for instance, fixed effects or random effects models in future research.

Our main contribution to the literature is the identification of longitudinal associations between participation in organized leisure sport and social well-being in school. We assessed well-being using a validated standardized measure employed across all Danish public schools as part of mandatory annual assessments. Specifically, our index measured school-related social well-being. We hypothesized that a relationship might exist because more than 80 percent of children between the ages of 7–15 participate in sports or exercise [35]. In total, 82 percent of these play sports in a sports association [35]. In the Danish context, most leisure-time sport activities are organized in local organizations and sports associations. The majority of children who are active in sports are very likely to engage in sports with peers from their own school or class. Significant overlap between the social networks at sport and at school thus exists for many children. While we cannot empirically investigate the potential mechanisms at play, other studies suggest that strengthened social relationships are key mechanisms for generating well-being through sport [21,67]. The overlap of social networks in sport and school, and the development of positive social relationships across these arenas could potentially explain why we observe a spillover. An additional potentially relevant mechanism could be self-assessed athletic competence [21]. Self-assessed athletic competence refers to how good you believe you are at sports in general or at a specific sport. If your self-assessment is correct, you are more prone to continue in sports, might be praised for your athleticism, and develop positive social relationships with peers. Due to the overlap of social networks in sports and school in Denmark, this could potentially reinforce school-related social well-being. While these explanations are theoretically plausible, they have not been measured or tested as part of our study. Further research could therefore focus on the exploration of mechanisms.

Controlled trials are the gold standard for estimating effects. However, conducting trials is not possible in relation to voluntary participation in leisure-time sports. For that reason, we call for additional studies employing quasi-experimental approaches on longitudinal datasets focusing on sports participation as it unfolds in non-artificial, non-clinical real-world settings. In particular, we see a need for more studies exploring and testing the social mechanisms that potentially bring about changes in youth well-being in voluntary non-clinical settings. The focus of this article has been on sports participation as a social activity rather than the benefits of physical exercise and the frequency and the level of competition. However, the conclusion is limited due to a lack of information on the frequency of training, as more time spent training could imply more time in a positive social milieu. As a result, the study captures the average association of sports participation and does not allow us to distinguish whether the observed associations vary according to the intensity, frequency, or level of involvement in sports activities. Issues of self-selection and unobserved confounding need to be carefully handled in future research.

## 5. Conclusions

Our results showed that voluntary participation in organized sports was positively associated with subsequent school-related social well-being levels in a Danish sample of fifth- to ninth-grade pupils. Cohen’s d was 0.31, which is similar to differences found in meta-analyses on the subject. The main contribution to the literature is the identification of longitudinal observational associations found in a non-clinical setting, and the finding that leisure-time sports are associated with well-being in school. Future research should use longitudinal research designs that explicitly model repeated observations, changes in sports participation over time, and time-varying confounding.

## Figures and Tables

**Table 1:** Distributions of responses in the three waves of surveys among pupils.

	Wave 1	Wave 2	Wave 3
Sex			
Boys	265 (49.6%)	443 (47.6%)	376 (50.8%)
Girls	269 (50.4%)	487 (52.4%)	364 (49.2%)
Grade			
5th grade	187 (35.0%)	-	-
6th grade	194 (36.3%)	281 (30.2%)	165 (22.3%)
7th grade	117 (21.9%)	243 (26.1%)	261 (35.3%)
8th grade	36 (6.7%)	213 (22.9%)	157 (22.1%)
9th grade	-	193 (20.8%)	157 (23.0%)
Total (100%)	534	930	740

**Table 2:** Descriptives of the treatment variable and the outcome variable.

	Activity Status, n in Parentheses (2021–2022)	2020 Mean Well-Being Score Sdt. Deviation in Parentheses	2022 Mean Well-Being Score Sdt. Deviation in Parentheses
No sport (inactive)	27.6% (276)	4.10 (0.69)	3.95 (0.63)
Sport participant (active)	72.4% (723)	4.28 (0.54)	4.14 (0.56)
Total	100% (999)	4.23 (0.59)	4.09 (0.59)

**Table 3:** AIPW estimates. The association between leisure-time sports participation and school-related social well-being. Standard errors are in parentheses.

	Association with Subjective Social Well-Being	95% Confidence Interval
Impact of sports participation (n = 998)	0.180 *** (0.045)	[0.091–0.268]
Impact of sports participation, including only respondents who were included in the panel after Wave 1 (557)	0.155 ** (0.059)	[0.040–0.271]
Impact of sports participation, including prior levels of well-being in the model (n = 586)	0.163 ** (0.052)	[0.061–0.265]
Impact of sports participation, including only respondents who did not change their sports participation status (n = 606)	0.235 *** (0.060)	[0.118–0.352]

* = 0.05. ** = 0.01 *** = 0.001.

**Table 4:** Exploratory AIPW analyses of prolonged exposure to organized sports. Standard errors in parentheses.

Respondents Who Answered All Waves (n = 223)	
Sports active in 1 to 2 waves	0.129 (0.102)
Sports active in all the waves	0.185 * (0.089)
Respondents who answered Wave 3 and *at least* one more wave (n = 486)	
Respondents who did not change their sports participation status	0.240 *** (0.070)

* = 0.05. ** = 0.01 *** = 0.001.

## Data Availability

The datasets presented in this article are not readily available because Danish register data has been used for the study. These register data (micro data) are located on secure servers at Statistics Denmark. It is strictly prohibited to share or download these data. Our own three waves of survey data are available upon request.

## Supplementary Materials

The following supporting information can be downloaded at https://www.mdpi.com/article/10.3390/healthcare14193288/s1. Figure S1: Flow diagram depicting the process from initial sample to analytical sample. Figure S2: Overlap plot. Figure S3: Overlap plot for respondents who answered all waves of the survey. Table S1: Logistic regression, with sports participation as the dependent variable. Std. Errors in parentheses. Table S2: Covariate balance summary without prior school-related social well-being. Table S3: Covariate balance summary including prior school-related social well-being.
