# Supplementary material for: The Longitudinal Association Between Organized Leisure Sports and School-Related Social Well-Being
Source: Healthcare (Basel). 2026 Oct 2;14(19):3288. doi: 10.3390/healthcare14193288 (PMC13665283; doi:10.3390/healthcare14193288)
Supplement: Supplementary file 1 [file healthcare-14-03288-s001.zip › healthcare-4421299-supplementary.pdf]

Figure S1: Flow diagram depicting the process from initial sample to analytical sample.

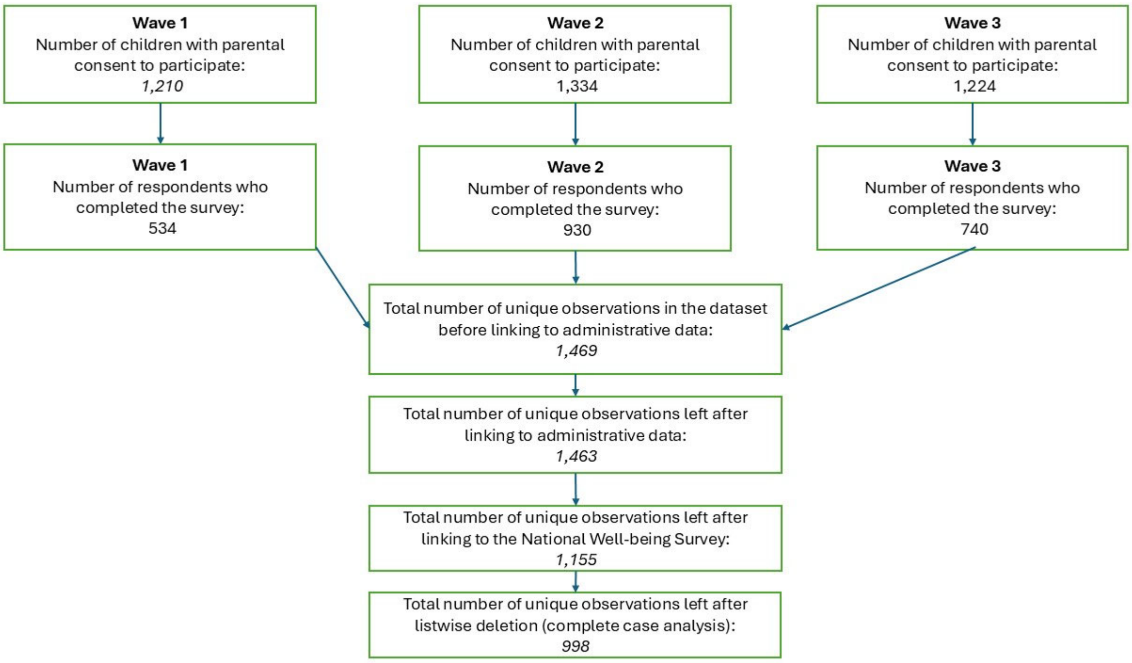

Figure S2: Overlap plot

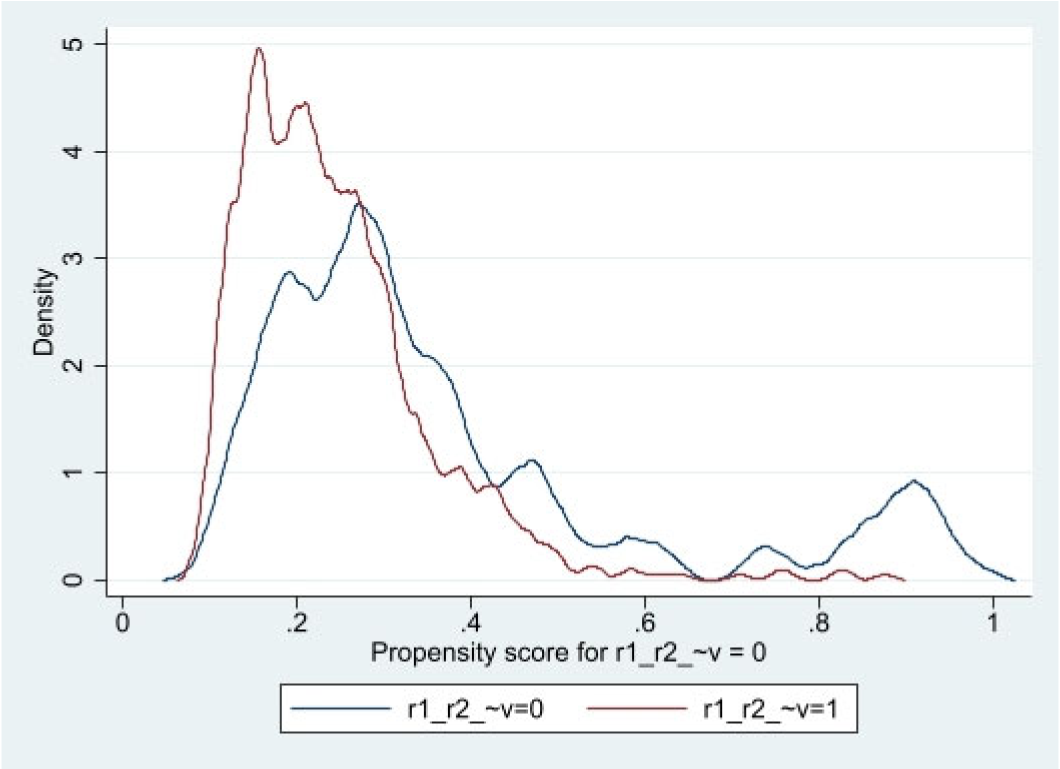

Table S1: Logistic regression, Sports participation as the dependent variable. Std. Errors in parentheses

	Coefficient
**2020 School-related social well-being**	1.58*** (0.20)
**2019 employment rate (own)**	0.47 (0.27)
**2019 employment rate (mother)**	1.05** (0.02)
**2019 employment rate (father)**	1.01 (0.02)
**Educational attainment (mother)**	
Unskilled mother	Re[illegible]
Skilled	0.945 (0.23)
Further education	1.12 (0.28)
**Educational attainment (father)**	
Unskilled mother	Re[illegible]
Skilled	1.36 (0.27)
Further education	1.64* (0.38)
**Gender (ref=male)**	1.01 (0.17)
**Year of birth**	1.29*** (0.08)
**Family Composition**	
Living with both parents	Re[illegible]
Lives with mother, who is in a relationship	0.71 (0.18)
Lives with a single mother	0.67 (0.15)
Lives with [illegible]ather, who is in a relationship	0.305* (0.17)
Lives with a single [illegible]ather	0.61 (0.23)
Lives with neither parent	1.98 (1.25)
**2019 Number of children in the household**	0.03 (0.02)
**Pseudo r-squared**	0.063
**N**	993

Table S2: Covariate balance summary without prior school related social well-being

	Standardized differences		Variance ratio	
	Raw	Weighted	Raw	Weighted
**Mother's mean hours of employment**	0.227	0.021	0.810	0.955
**Father's mean hours of employment**	0.162	0.036	0.927	1.062
**Mother's mean number of weeks on disability benefit**	-0.093	-0.017	0.554	0.873
**Father's mean number of weeks on disability benefit**	-0.192	-0.018	0.188	0.781
**Not living with both parents**	-0.262	-0.023	0.812	0.979
**Number of children in the household**	-0.083	0.016	0.732	1.002
**Mother's educational attainment**				
**Vocational education**	-0.152	-0.002	0.918	0.998
**Further education**	0.242	0.019	1.024	1.000
**Father's educational attainment**				
**Vocational education**	-0.039	0.009	0.996	1.001
**Further education**	0.205	0.000	1.200	1.000
**Gender**	-0.043	-0.011	0.998	1.000
**Year of birth**	0.380	0.003	0.864	1.012
**Prior sports participation**	-0.475	-0.053	0.065	0.754

Table S3: Covariate balance summary including prior school related social well-being

	Standardized differences		Variance ratio	
	Raw	Weighted	Raw	Weighted
**Mother's mean hours of employment**	0.085	0.019	0.936	0.955
**Father's mean hours of employment**	0.128	0.050	0.978	1.034
**Mother's mean number of weeks on disability benefit**	0.037	0.019	1.173	1.048
**Father's mean number of weeks on disability benefit**	-0.050	0.002	0.538	0.940
**Not living with both parents**	-0.212	-0.032	0.845	0.973
**Number of children in the household**	-0.182	-0.017	0.831	1.072
**Mother's educational attainment**				
**Vocational education**	-0.201	-0.023	0.890	0.984
**Further education**	0.129	0.021	0.988	0.996
**Father's educational attainment**				
**Vocational education**	-0.054	-0.021	0.996	0.999
**Further education**	0.179	0.035	1.139	1.024
**Gender**	-0.016	-0.022	0.997	1.000
**Year of birth**	0.279	-0.024	0.780	0.920
**Prior sports participation**	-0.466	-0.062	0.070	0.730
**School related social well-being in 2019**	0.181	0.057	0.852	1.039

Figure S3: Overlap plot for respondents having answered all waves of the survey

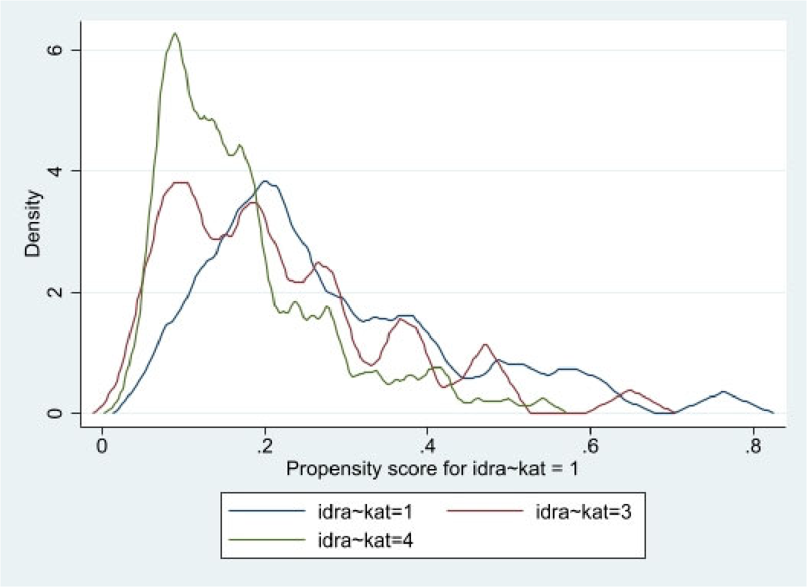

Density
0
2
4
6
0
.2
.4
.6
.8
Propensity score for idra~kat = 1
idra~kat=1
idra~kat=3
idra~kat=4
